# Relationships Among Cognitive Function, Frailty, and Health Outcome in Community-Dwelling Older Adults

**DOI:** 10.3389/fnagi.2021.790251

**Published:** 2022-01-21

**Authors:** Huiping Xue, Chunxia Huang, Qin Zhu, Shuixin Zhou, Yunlan Ji, Xiaohui Ding, Dandan Zhang, Dongmei Gu

**Affiliations:** ^1^Emergency Intensive Care Unit, Affiliated Hospital of Nantong University, Nantong, China; ^2^Nursing Department, Affiliated Hospital of Nantong University, Nantong, China

**Keywords:** cognitive impairment, frailty, quality of life, activities of daily living, social activities

## Abstract

**Background:**

Frailty and cognitive impairment are significant problems faced by older adults, which have a significant impact on their activities of daily living, social activities, and quality of life.

**Design:**

Cross-sectional study.

**Methods:**

A total of 252 older adults in two communities in Yangzhou were randomly selected. The cognitive function of the elderly was assessed using the Memory and Executive Screening (MES). The frailty phenotype was used to evaluate the frail situation of older adults. The activity of daily living (ADL), functional activities questionnaire (FAQ), and European quality of 5-dimensions (EQ-5D) were used to evaluate health outcomes in the elderly. SEM was used to explore the direct and indirect relationship among cognitive function, frailty and health outcomes.

**Results:**

There was a significant direct correlation between cognitive function and frailty; the direct effect was −0.521. The influence path of cognitive function on health outcomes included direct and indirect effects; the total effect was −0.759. The effect of frailty on health outcomes included direct and indirect effects; the total effect was 0.440.

**Conclusion:**

According to SEM, cognitive function interacts with frailty and may reduce the quality of life, the ADL, and social activities among older adults directly and indirectly, so future assessments of older adults should consider both cognitive function and frailty, so as to further improve the health outcome of the elderly. When formulating relevant intervention measures in the future, we need to consider that it cannot only improve the cognitive function, but also improve the frail situation, so as to jointly improve the health outcomes of older adults.

## Introduction

By the end of 2018 the population ≥ 60 years of age in China accounted for 17.90% of the total population, thus indicating that the degree of aging in China is on the rise (National Bureau of Statistics of China, 2019). Cognitive impairment and frailty are the most common geriatric syndromes in older adults, which pose a major threat to them, as specifically reflected in the aggravation of disability, a decline in the quality of life, and an increase in mortality (Fabrício et al., 2020). Cognitive impairment generally refers to various degrees of cognitive dysfunction caused by various reasons, including various stages from mild cognitive impairment (MCI) to Alzheimer’s disease (AD). Cognitive impairment and frailty often occur in the same older adults and interact with each other. The coexistence of the two can accelerate the decline of physical and cognitive functions and form a vicious cycle (Brigola et al., 2015). Frailty refers to a weakening in strength and a disorder in physiological function, which will lead to an increase in dependency, vulnerability, and susceptibility to death (Dent et al., 2017). Older adults with frailty have a reduced ability to cope with acute diseases, and have a correspondingly increased risk of falls, disability and death (Fried et al., 2001). The study found that the risk of death, hospitalization, disability and fall in older adults with frailty was 1.7–4.4 times higher than those without frailty (Zheng et al., 2016).

### Relationship Between Cognitive Function and Frailty

Aging is associated with physical frailty and cognitive decline. Frail older adults are at higher risk for cognitive decline (Calderón-Larrañaga et al., 2019; Grande et al., 2019). The studies showed that frailty was significantly correlated with the incidence of cognitive impairment (Armstrong et al., 2016; Tsutsumimoto et al., 2019), and the incidence of cognitive impairment in frail older adults was 8 times that of normal older adults (Kulmala et al., 2014). Meanwhile, frailty predicts a poorer cognitive development trajectory among MCI patients, and is associated with the high risk of developing MCI (Kiiti Borges et al., 2019). In addition, based on data from the Canadian Study of Health and Aging, Song et al. (2014) found that the incidence rate of cognitive impairment increased exponentially with the increase of frailty index, and frailty is an independent risk factor for cognitive impairment. Cognitive impairment is related to frailty, and both have common biological mechanisms, including genetic alternations, immune system dysfunction, and neuroinflammation (Sargent et al., 2020).

### Effects of Cognitive Function and Frailty on Health Outcomes

Studies have shown that frailty interacts with cognitive impairment, which leads to adverse health outcomes, such as a poorer quality of life (Feng et al., 2017). Hussenoeder et al. (2020) showed that the quality of life in older adults with MCI is closely related to cognition and is lower than healthy older adults. Cognitive function can affect the ADL, social activities, and quality of life among older adults (Ginsberg et al., 2019). Frailty increases the risk of adverse health outcomes, such as falls, disability, decreased ADL, limited physical activity, falls, risk of admission to the hospital and death (Vermeiren et al., 2016; Panza et al., 2018; Zhang et al., 2019). Kojima (2018) showed that decreased ADL in frail older adults is significantly higher than that in healthy older adults. Meanwhile, Audai et al. (2020) found that frail older adults have a lower quality of life than non-frail older adults. Although MCI and frailty interact with each other, cognitive function and frailty affect ADL, social activities, and the quality of life, but a study of the interaction and influence has not been reported.

Structural equation modeling (SEM) is a method to establish, estimate and test causality models. It can study not only the explicit variables, but also the latent variables, and it can display the relationship and size of variables through path graph (Wu, 2010). Therefore, this study intends to use SEM to explore the direct and indirect relationship among cognitive function, frailty and health outcomes, so as to provide reference for the later development of intervention measures to improve the health level and quality of life of the older adults in the community.

## Materials and Methods

### Participants

In general, the minimum sample size is required to be more than 200 to obtain a relatively stable model. Some scholars believe that the sample size should be 5–10 times of the free parameter to be estimated before it is considered acceptable (Kline, 2005; American Psychological Association, 2010). Therefore, a total of 252 older adults who lived in 2 Yangzhou City communities from September 2017 to June 2018 were selected using the random sampling and random number methods. The exclusion criteria were as follows:(1) older adults with mental disorders, such as depression and an unstable condition; (2) older adults with severe hearing and vision disorders who could not cooperate; (3) older adults with language communication disorders who could not complete the investigation; (4) older adults with central nervous system damage caused by severe diseases, such as tumors and infections; (5) older adults with cerebrovascular disease and an unstable condition; and (6) older adults who were unwilling to sign informed consent.

### Cognitive Assessment

All subjects completed the MES. MES was developed by Guo et al. (2012) for the screening of AD and MCI, including memory factor and executive factor, with 50 points for each part. The higher the score, the better the cognitive function, and when the score is less than 72, amnestic MCI can be diagnosed. The memory test adopts immediate recall, short delayed recall and long delayed recall, and the executive function test mainly includes fluency, finger 1 test, visuospatial structure ability and finger 2 test, which can effectively reflect the major cognitive impairment areas (Guo et al., 2012). The correlation coefficients of MES-M and MES-E with MES were 0.89 and 0.88, and the intra-group correlation coefficients were 0.92 when tested again at an interval of 23–35 days, suggesting that MES has good reliability and validity (Guo et al., 2012). MES is simple to operate and execute, and is not affected by education level, and it is time-consuming and can quickly assess the degree of impairment of episodic memory and executive function in major cognitive areas without obvious ceiling and floor effects.

### Frailty Assessment

Frailty phenotype assessment was proposed by Fried et al. (2001), including 5 items, which are (1) body mass decline: in the past year, body mass decline > 4.54 kg or > 5% of body mass; (2) Slow walking speed: measure the time required to walk 4 m, and judge whether the walking speed is slow according to height and gender, it involves measuring the 4-m distance on the open ground in advance, to instruct the older adults to walk normally as usual, and record the time with a stopwatch; (3) Grip strength weakening: use the grip dynamometer to measure the grip strength, and the patient was told to measure the hand strength twice with the dominant hand to get the maximum value, and whether the grip strength was weakened was judged according to gender and body mass index; (4) Low physical activity: use the international physical activity scale (Liou et al., 2008) to calculate the amount of exercise within 1 week. When the amount of exercise for men and women is less than 383 and 270 kcal, respectively, the amount of physical activity is low; (5) Fatigue: the items of self-rating depression scale were asked: “do you feel that you have to make efforts to do everything in the past week, which has occurred for several days” and “can’t walk forward in the past week, which has occurred for several days” are used for inquiry. If one item lasts for more than 3 days, it is fatigue (Geriatrics Branch of the Chinese Medical Association, 2017). The value of each item is 1 point, 1 point will be counted if the item is satisfied, otherwise, no score will be scored. According to the frailty phenotype score as the gold standard, when the score is 0∼2, it is the non-frailty stage, when the score is ≥ 3, it is the frailty stage (Fried et al., 2001).

### Health Outcome Assessment

The ADL was developed by Lawton and Brody (1969), which is composed of two parts, basic activities of daily living (BADL) and instrumental activities of daily living (IADL). There are 14 items in the scale, and the score of each item ranges from 1 to 4 points. If 2 or more items are ≥ 3, or the total score is ≥ 22 points, it indicates that the ability of daily living is significantly reduced. The FAQ was compiled by Pfeffer et al. (1982), with a total of 10 items, such as correct use of various tickets, timely payment of various bills, and self-shopping. The score was 0–3 points, with a total score of 0–30 points. The higher the score, the worse the social function. The EQ-5D was used to describe the QoL and health outcome (Gottschalk et al., 2020). It includes five dimensions: mobility, self-care, usual activities, pain/discomfort, anxiety/depression. Each dimension has three levels, and respondents can make choices on five dimensions and three levels in the questionnaire, and calculate EQ-5D index scores through the utility value conversion table. The EQ-5D is the most widely used QoL assessment scale worldwide.

### Quality Control

Before the survey, professional physicians conducted standardized training for team members, including subject-related professional knowledge, scale evaluation and survey terminology. After the training, the study can be carried out only after passing the assessment. The cognitive function of the subjects was assessed by the same neurologist. All surveys were conducted face-to-face in the community meeting room to ensure that there was no interference in the survey process. The final completed questionnaire was confirmed by two people to ensure the integrity of the questionnaire. Data was performed by two persons to ensure the correctness of data entry.

### Statistical Analyses

All survey data were recorded by two persons using Excel software and SPSS 24.0 was used for statistical analysis. The measurement data are expressed in the mean ± standard deviation ($\bar{\text{x}}\pm s$). The exploratory factor analysis was carried out using SPSS 22.0 statistical software. The confirmatory factor analysis and SEM were established using AMOS21.0 statistical software. *P* < 0.05 was statistically significant.

## Results

### Baseline Characteristics

There were 252 older adults in the communities, 82 of whom had MCI (prevalence rate = 32.54%). In terms of age distribution, the age of the subjects ranged from 60 to 89 years old, with an average age of (70.76 ± 7.88) years old. In terms of gender distribution, the proportion of men and women is balanced. The majority of older adults had hypertension, other variables such as education level, occupation, marital status and BMI are shown in Table 1.

**TABLE 1:** Characteristics of participants (*n* = 252).

Characteristics	Frequency	Proportion(%)
**Gender**		
Male	128	50.79
Female	124	49.21
**Educational level**		
Primary and below	54	21.43
Junior high	95	37.70
Senior high and above	103	40.87
**Occupation**		
Mental labor	115	45.63
Physical labor	137	54.37
**Marital status**		
Married	208	82.54
Divorced/Widowed	44	17.46
**Hypertension**		
No	120	47.62
Yes	132	52.38
**Diabetes**		
No	209	82.94
Yes	43	17.06
**Coronary heart disease**		
No	232	92.06
Yes	20	7.94
**Cerebral infarction**		
No	245	97.22
Yes	7	2.78
**Hyperlipidemia**		
No	217	86.11
Yes	35	13.89
**Family history of dementia**		
No	233	92.46
Yes	19	7.54
**BMI**		
18.5	10	3.97
18.5∼23.9	108	42.86
24∼27.9	98	38.89
28	36	14.29

### Cognitive Function, Frailty, and Health Outcome Scores

Based on the assessment of cognitive function, frailty, and health outcomes of the elderly in the communities, it was shown that there is a huge range of scores in the assessment of them. The overall cognitive function score was 80.57 ± 8.91; the memory function score was 36.15 ± 6.70 and the executive function score was 44.41 ± 4.42. The total frailty score was 0.37 ± 0.65; the healthy outcome score was 14.50 ± 1.03 for ADL, 2.08 ± 2.55 for the FAQ, and 0.21 ± 0.06 for the EQ-5D; Table 2).

**TABLE 2:** Scores of cognitive function, frailty, and health outcome (*n* = 252).

Item	Minimum	Maximum	$\bar{\text{x}}\pm s$	95%CI
MES-T	57	97	80.57 ± 8.91	79.46, 81.67
MES-M	12	48	36.15 ± 6.70	35.29, 37.02
MES-E	32	50	44.41 ± 4.42	43.86, 44.96
ADL	14	18	14.50 ± 1.03	14.37, 14.63
FAQ	0	8	2.08 ± 2.55	1.76, 2.39
EQ-5D	0.15	0.42	0.21 ± 0.06	0.20, 0.22
Frailty phenotype	0	2	0.37 ± 0.65	0.29, 0.45
Grip strength (kg)	9.60	47.60	29.08 ± 7.97	28.09, 30.07
4 m Walking time (s)	2.60	8.50	4.36 ± 0.79	4.26, 4.46

*MES-E, MES Executive; MES-M, MES Memory; ADL, Activity of Daily Living Scale; FAQ, Functional Activities Questionnaire; EQ-5D, European Quality of 5-Dimensions.*

### Relationships Between Cognitive Function, Frailty, and Health Outcome

Table 3 documents the results of correlation analyses of cognitive function, frailty, and health outcome. The results of the Pearson’s correlation analyses showed that MES-M was significantly correlated with MES-E, ADL, FAQ, self-reported exhaustion and grip strength (*P* < 0.05). MES-E was significantly correlated with ADL, FAQ, self-reported exhaustion (*P* < 0.05). ADL was significantly correlated with FAQ and grip strength (*P* < 0.05). FAQ was significantly correlated with self-reported exhaustion and grip strength (*P* < 0.05). Also, self-reported exhaustion was significantly correlated with grip strength (*P* < 0.05).

**TABLE 3:** Pearson’s correlation coefficient of study variables (*n* = 252).

	1	2	3	4	5	6	7	8	9
MES-M	1								
MES-E	0.173**	1							
ADL	−0.179**	−0.155*	1						
FAQ	−0.301**	−0.231**	0.452**	1					
EQ-5D	–0.019	–0.058	0.088	0.079	1				
Physical activity	0.039	–0.062	–0.009	0.067	0.005	1			
Self-reported exhaustion	−0.129*	−0.137*	0.066	0.181**	0.072	0.079	1		
4 m walking time	–0.006	–0.115	0.076	–0.042	–0.034	0.052	–0.057	1	
Grip strength	0.222**	0.063	−0.177**	−0.277**	–0.069	–0.074	−0.292**	−0.044	1

*P < 0.05, **P < 0.01.

### Explorative Factor Analyses

#### Item Discriminant Validity Analyses

The size of the correlation between the test variables is the premise of explorative factor analysis. The judgment indicators mainly included the Kaiser-Meyer-Olkin (KMO) value and Bartlett’s test of sphericity. The KMO values were between 0 and 1. The larger the value, the better the result of factor analysis. A KMO value < 0.5 was not suitable for factor analysis (Wu and Pan, 2014). In the current study, the KMO value was 0.631 > 0.500 and the Bartlett test of sphericity χ^2^ was 295.105 (*P* < 0.001); the difference was statistically significant, indicating that there was a strong correlation between the variables, and suitable for factor analysis.

### Factor Analyses

Principal component analysis (PCA) was used to calculate the correlation matrix, eigenvalues, and eigenvectors among the variables. The eigenvalues were arranged from large-to-small to calculate the corresponding principal components.

The matrix of initial components was rotated by the maximum variance orthogonal rotation method, and the final factor loading matrix was obtained. There were three common factors with eigenvalues > 1. The first common factor was the total MES score, which is used to evaluate cognitive function, so this factor is referred to as cognitive function. The second common factor was ADLs, social activities, and QoL were similar, which was referred to as health outcomes. The frailty phenotype score was third common factor, which was referred to as frailty. The three common factors finally accounted for 65.431% of the total variation (Supplementary Tables 1, 2).

### Construction of Structural Equation Modeling, Model Modification and Fit Change

The model was a non-recursive model, which had 10 observation variables and 33 parameters to be estimated. According to the *t* rule [33 < 10 × (10 + 1)/2], the model was identified. The maximum likelihood (ML) method was selected as the model estimation method.

**FIGURE 1:**
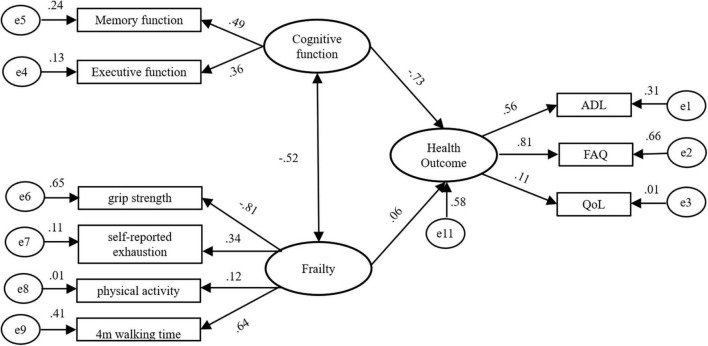
A path diagram of direct and indirect influences of cognitive function, frailty and health outcome among community-dwelling elderly people.

The fitting degree test of the initial model indicated that the fitting result was not ideal, so it was necessary to modify the initial model using a parameter test and correction index to achieve good fitting. Amos provided two model correction indices, including a modified index (MI) and critical ratio (CR) (Fang et al., 2018). In the process of model fitting, professional knowledge is also required. According to the CR (*t*-value) provided by Amos, model parameters with no significant difference were deleted. The results showed that body weight had no effect on frailty (*P* = 0.132 > 0.05), so this item was deleted, which was consistent with domestic and international studies (Moreira and Lourenço, 2013; Hou et al., 2018). The final structural equation model was determined and the modified model is shown in Figure 1.

Through the modified fitting, the overall fitness of the model reached a good state, and each fitting index also showed that the model fitted well (Table 4).

**TABLE 4:** Model fit indices.

Index	Acceptable range	Value
χ^2^-value		26.790
*P-*value	>0.050	0.314
RMSEA	<0.050	0.022
GFI	>0.900	0.978
AGFI	>0.900	0.958
CFI	>0.900	0.988
TLI	>0.900	.982

*RMSEA, Root Mean Square Error of Approximation; GFI, Goodness of Fit Index; AGFI, Adjusted Goodness of Fit; CFI, Comparative Fit Index; TLI, Tucker-Lewis Index.*

### Factor Path Analyses

Table 5 shows the path and path coefficients of the cognitive function effects on health outcomes and frailty. It can be seen from Table 5 that the interaction between cognitive function and frailty was a direct effect, and the path coefficient was −0.521, indicating that the better the cognitive function, the less likely frailty occurs. The influence of cognitive function on health outcomes included direct and indirect effects. There was one path of a direct effect with a path coefficient of −0.728, and there was one path coefficient with an indirect effect with a path coefficient of −0.031. The total effect of cognitive function on health outcomes was −0.759, indicating that the better cognitive function, the lower the health outcome score. The influence of frailty on health outcomes included direct and indirect effects. There was one path of a direct effect with a path coefficient of 0.060 and one path coefficient of an indirect effect with a path coefficient of 0.380. The total effect of decline on health outcome was 0.440, which indicated that the more severe the frailty, the worse the health outcomes (Table 5).

**TABLE 5:** Pattern effect results of cognitive function, frailty, and health outcome.

Influence path	Direct effect	Indirect effect	Total effect
Frailty↔ Cognitive function	–0.521		–0.521
Health outcome←Cognitive function	–0.728		–0.728
Health outcome←Frailty←Cognitive function		–0.031	–0.759
Health outcome←Frailty	0.060		0.060
Health outcome←Cognitive function←Frailty		0.380	0.440

## Discussion

Studies have shown that the frail older adults have a higher risk of MCI (Borges et al., 2019), and the ADL and quality of life will decline (Audai et al., 2020). At the same time, the ADL, social activities, and QoL of the elderly with MCI will also be affected (Ginsberg et al., 2019), but the study on the interaction and connection between the three has not been demonstrated. Therefore, based on explorative analysis, SEM was applied to construct a cognitive intervention model to provide support for later intervention. The results suggested that cognitive function, frailty and health outcomes are closely related. Specifically, cognitive function interacts with frailty and may reduce the quality of life, the ADL, and social activities among the elderly. Through modification, a well-fitted model was created, and the pathways and total effects of cognitive function on frailty and health outcomes are listed in the results.

### Relationship Between Cognitive Function and Frailty

The results of path analysis showed that cognitive function and frailty interacted with each other, and the path coefficient was −0.521, which was consistent with international studies. De Cock et al. (2018) reported that frailty is associated with the decline in cognitive function, which is accompanied by accelerated aging. Grip strength and fatigue are the main causes of frailty in the elderly (Audai et al., 2020). Symeon et al. (2018) found that fatigue, as a sign of physical frailty, is related to cognitive function and can be used as a supplementary indicator of cognitive assessment. Grip strength is one of the manifestations of muscle loss, which can be used as a predictor of cognitive decline with age (Fritz et al., 2017).

### Effects of Cognitive Function on Health Outcomes

In this study, the effect of cognitive function on health outcomes was −0.759, which was a significant effect. Altieri et al. (2021) found that compared with healthy older adults, the elderly with MCI had more difficulties with ADL, especially instruments of activities of daily living (IADL). Social activities require the participation of multiple cognitive domains, therefore, social activities of the elderly with MCI are also significantly reduced (Becker et al., 2021). Hussenoeder et al. (2020) found that QoL is closely related to cognitive function, and QoL of with MCI older adults is lower than healthy elderly. Cognitive impairment is the direct cause of the decline in the quality of life of the elderly (Marshall et al., 2011). Cognitive impairment, particularly those related to memory and executive function, can prevent patients from performing some activities of daily living. The main driving factors of the quality of life are: full of energy, pain free, ability to carry out activities of daily living and to move around (Salkeld et al., 2000; Molzahn et al., 2010). For MCI older adults, complex ADL is the most affected in the activities of daily living, while basic activities of daily living are often maintained to the AD stage. One of the main drivers of quality of life in older adults is the ability to carry out activities of daily living. Therefore, cognitive impairment can also affect the quality of life of the elderly.

In addition, the current study showed that cognitive function not only directly affected health outcomes, but also indirectly affected health outcomes through frailty. The quality of life (Dong, 2017) and ADL (Kojima, 2018) of the frail elderly will decline. With the increase of age, physical frailty (poor ability to perform daily living activities), psychological frailty (loneliness) and social frailty (social relationships) may occur to varying degrees due to the influence of personal factors and disease factors, which will eventually lead to the decline of quality of life (Gobbens et al., 2010). Fatigue is generally considered to be a key factor of frailty, and muscle fatigue (assessed by continuous grip strength) is associated with quality of life (Rizzoli et al., 2013). As a manifestation of muscle loss, grip strength is associated with cognitive function, which further affects quality of life.

### Effects of Frailty on Health Outcomes

In the current study, the direct effect of frailty on health outcomes was 0.060, which was significantly less than the indirect effect, possibly because the decline in quality of life and ADL is not the main manifestation of frailty (Xi and Guo, 2014). Moreover, this study was conducted in the community. The quality of life and daily living ability of the elderly in the community are acceptable. Therefore, the direct effect of frailty on the health outcomes of the elderly in the community is not so significant. In the future, the impact of frailty on the health outcomes of the elderly in the communities and nursing institutions can be considered. In addition, there are many ways for health outcomes, including psychological status and sleep status. Frail older adults are more prone to depression (Soysal et al., 2017). Insomnia is far more common in frail older adults than in non-frail older adults (Lee et al., 2018), and sleep disorders are more likely to occur. In addition, the frail older adults are more likely to suffer from diseases, pain and taking a variety of drugs which will also reduce their sleep quality (Du, 2017). All of the above reasons may lead to the direct effect of frailty on health outcomes to be smaller than the indirect effect.

There are many other health outcomes for the elderly. In this study, health outcome as an endogenous potential variable has a measurement error of 0.58, indicating that the part of health outcomes that could not be explained by cognitive function and frailty was an error in the SEM. This suggests that we can expand the health outcomes of the elderly in the future, such as psychological status and sleep quality, so as to further improve the SEM. In this study, it was found that cognitive function can affect health outcomes in the elderly, and it can also affect health outcomes in the elderly by influencing frailty. Similarly, frailty can affect health outcomes in older adults, as well as by affecting cognitive function. Frailty and cognitive impairment are common geriatric syndromes, so future assessments of older adults should consider both cognitive function and frailty, so as to further improve the health outcome of the elderly. At the same time, we can explore the intervention methods suitable for cognitive impairment and weakness, so as to improve the quality of life of the elderly. At the same time, we can explore appropriate interventions for cognitive impairment and frailty to improve the health outcomes of the elderly.

## Conclusion

In this study, SEM was used to explore the relationship among cognitive function, frailty and health outcomes among the elderly in the community. This model has a good fitting degree. When formulating relevant intervention measures in the future, we need to consider that it cannot only improve the cognitive function of the elderly, but also improve the frail situation, so as to jointly improve the health outcome of the elderly. In addition, there are other manifestations of health outcomes in the elderly, and we need to consider the impact of this aspect in future studies to improve the SEM. Moreover, although the model has a good degree of fitting, due to the limitation of sample size, the impact of the increase of data on the model needs to be verified in the later stage. And it would be great to test the model by a subset of data from new samples. Since this study was only carried out in Yangzhou community and did not include the elderly in hospital or in nursing institutions, the sample lacks certain representativeness, which can be further improved in the future.

## Data Availability Statement

The raw data supporting the conclusions of this article will be made available by the authors, without undue reservation.

## Ethics Statement

The studies involving human participants were reviewed and approved by the Ethics Committee of Northern Jiangsu People’s Hospital. The patients/participants provided their written informed consent to participate in this study.

## Author Contributions

HX drafted the manuscript and performed statistical analyses. CH, YJ, XD, and DZ collected the data. QZ and SZ controlled the quality of data. DG and HX conceived and designed this research. All authors edited and approved the final manuscript.

## Conflict of Interest

The authors declare that the research was conducted in the absence of any commercial or financial relationships that could be construed as a potential conflict of interest.

## Publisher’s Note

All claims expressed in this article are solely those of the authors and do not necessarily represent those of their affiliated organizations, or those of the publisher, the editors and the reviewers. Any product that may be evaluated in this article, or claim that may be made by its manufacturer, is not guaranteed or endorsed by the publisher.
