# Supplementary material for: Relationships Among Cognitive Function, Frailty, and Health Outcome in Community-Dwelling Older Adults
Source: Front Aging Neurosci. 2022 Jan 21;13:790251. doi: 10.3389/fnagi.2021.790251 (PMC8814599; doi:10.3389/fnagi.2021.790251)
Supplement: Supplementary file 1 [file Table_1.docx]

Supplementary Material

# Supplementary Tables

Supplementary Table 1. eigenvalues, variance percentage, and cumulative percentage of the factors

| Factor | Eigenvalue | % of Variance | Cumulative % of Variance |
| --- | --- | --- | --- |
| Cognitive function | 1.701 | 24.298 | 24.298 |
| Health outcome | 1.483 | 21.191 | 45.489 |
| Frailty | 1.396 | 19.942 | 65.431 |

Supplementary Table 2. Factor loading matrix after the method of maximum variance orthogonal rotation

| Name | Component | | |
| --- | --- | --- | --- |
|  | Cognitive function | Health outcome | Frailty |
| MES-T | 0.843 |  |  |
| ADL |  | 0.791 |  |
| FAQ |  | 0.726 |  |
| Quality of life |  | 0.495 |  |
| Frailty |  |  | 0.752 |

Abbreviations: MES-E, MES Executive; ADL, Activity of Daily Living Scale; FAQ, Functional Activities Questionnaire.

.
